# The Role of miR-103 and miR-107 in Regulation of *CDK5R1* Expression and in Cellular Migration

**DOI:** 10.1371/journal.pone.0020038

**Published:** 2011-05-23

**Authors:** Silvia Moncini, Alessandro Salvi, Paola Zuccotti, Gabriella Viero, Alessandro Quattrone, Sergio Barlati, Giuseppina De Petro, Marco Venturin, Paola Riva

**Affiliations:** 1 Department of Biology and Genetics for Medical Sciences, University of Milan, Milan, Italy; 2 Division of Biology and Genetics, Department of Biomedical Sciences and Biotechnology, University of Brescia, Brescia, Italy; 3 Centre for Integrative Biology (CIBIO), University of Trento, Trento, Italy; 4 Institute of Biophysics, National Research Council, Trento, Italy; Texas A&M University, United States of America

## Abstract

*CDK5R1* encodes p35, a specific activator of the serine/threonine kinase CDK5, which plays crucial roles in CNS development and maintenance. CDK5 activity strongly depends on p35 levels and p35/CDK5 misregulation is deleterious for correct CNS function, suggesting that a tightly controlled regulation of *CDK5R1* expression is needed for proper CDK5 activity. Accordingly, *CDK5R1* expression was demonstrated to be controlled at both transcriptional and post-transcriptional levels, but a possible regulation through microRNAs (miRNAs) has never been investigated. We predicted, within the large *CDK5R1* 3′UTR several miRNA target sites. Among them, we selected for functional studies miR-103 and miR-107, whose expression has shown a strong inverse correlation with p35 levels in different cell lines. A significant reduction of *CDK5R1* mRNA and p35 levels was observed after transfection of SK-N-BE neuroblastoma cells with the miR-103 or miR-107 precursor (pre-miR-103 or pre-miR-107). Conversely, p35 levels significantly increased following transfection of the corresponding antagonists (anti-miR-103 or anti-miR-107). Moreover, the level of *CDK5R1* transcript shifts from the polysomal to the subpolysomal mRNA fraction after transfection with pre-miR-107 and, conversely, from the subpolysomal to the polysolmal mRNA fraction after transfection with anti-miR-107, suggesting a direct action on translation efficiency. We demonstrate, by means of luciferase assays, that miR-103 and miR-107 are able to directly interact with the *CDK5R1* 3′-UTR, in correspondence of a specific target site. Finally, miR-103 and miR-107 overexpression, as well as *CDK5R1* silencing, caused a reduction in SK-N-BE migration ability, indicating that these miRNAs affect neuronal migration by modulating *CDK5R1* expression. These findings indicate that miR-103 and miR-107 regulate *CDK5R1* expression, allowing us to hypothesize that a miRNA-mediated mechanism may influence CDK5 activity and the associated molecular pathways.

## Introduction

Human *CDK5R1* (*Cyclin-dependent kinase 5*, *regulatory subunit 1*, GeneID: 8851) encodes p35, a specific activator of Cyclin-dependent kinase 5 (*CDK5*, GeneID: 1774), which is a proline-directed serine/threonine kinase. Monomeric CDK5 shows no enzymatic activity and requires association with p35 or p39 for activation [1]. While Cdk5 is expressed ubiquitously in mammalian tissues, its activator proteins are predominantly expressed in postmitotic neurons: for this reason its activity has mainly been associated with the central nervous system (CNS) development and function [2], [3].

The functions of Cdk5 that have been best characterized mainly involve CNS development and maintenance, and are mediated by the phosphorylation of its several substrates [2], [3].

The association of p35 with Cdk5 is required for neuronal survival and is involved in many fundamental processes during neuronal differentiation and function such as neurite outgrowth [4], axon regeneration [5], neuronal apoptosis [6], synaptic plasticity, learning and memory [7] and membrane trafficking during the outgrowth of neuronal processes [8], with a role also in secretion and dopamine signalling by controlling cytoskeletal dynamics [9].

Furthermore, Cdk5 and p35 are essential for radial migration and lamination of cortical neurons during the morphogenesis of the mammalian cerebral cortex. *Cdk5* knockout mice display severe cortical lamination defects and perinatal death [10]. Similarly, *Cdk5r1* KO mice show severe cortical lamination defects and suffer from adult mortality and seizures [11]. Cdk5 hyperactivation, associated to p35 overexpression and production of p25, a proteolytic fragment containing the C-terminal portion of p35 [12], has been implicated in some neurodegenerative disorders, such as Alzheimer's disease (OMIM: 104300) [13], Parkinson's disease (OMIM: 168600) [14] and amyotrophic lateral sclerosis (OMIM: 105400) [15]. More recently, *CDK5R1* has been indicated as a candidate for mental retardation in the NF1-microdeletion syndrome (OMIM: 162200) [16].

The deleterious effects of *CDK5* and *CDK5R1* dysregulation during both physiological and pathological processes strongly suggest that a precise spatio-temporal regulation of *CDK5R1* expression is needed for a proper activation of CDK5. It has been shown that p35 cellular level is the main limiting factor for the CDK5 kinase activity [17], but little is known about the regulation of p35 expression. Some data on the regulation of *CDK5R1* transcription have been reported. TNF-α, through activation of the ERK1/2 pathway, regulates *Cdk5r1* promoter activity in PC12 cells inducing a sustained and robust expression of *Cdk5r1*, thereby increasing Cdk5 kinase activity [18]. Moreover, IL-6 was found to affect p35 protein levels in hippocampal neurons, and IFN-γ increased p35 protein levels in neuroblastoma Paju cells [19], [20]. Some evidence of a regulation of p35 at post-translational level has also been reported: p35 was shown to undergo fast turnover through ubiquitylation and proteasomal degradation [21] and it has been recently reported that PKCδ phosphorylates p35, preventing its degradation in cultured cortical neurons and regulating the radial migration of layer II/III cortical neurons by stabilizing p35 [22].

We recently demonstrated that the expression of *CDK5R1* can be further modulated at post-transcriptional level by its 3′-UTR [23]. 3′-UTRs play key roles in post-transcriptional regulatory mechanisms, allowing a finely tuned spatio-temporal control of expression of several neuronal genes coding for growth-associated proteins [24], cytoskeletal elements [25], neurotransmitter biosynthetic enzymes and receptors [26], and also proteins associated to neurodegenerative disease such as the amyloid precursor protein [27]. Indeed, disturbances in post-transcriptional regulation can lead to neuronal dysfunction or, in extreme cases, to neuronal degeneration [28].

The *CDK5R1* gene displays a very large and highly evolutionary conserved 3′-UTR (2725 bp), where specific post-transcriptional regulatory elements/effectors, such as AU-rich regions and neuronal RNA-binding proteins ELAV (nELAV), were shown to affect transcript stability [23]. Nevertheless, other *trans*-acting factors are expected to have a role in the 3′-UTR-mediated modulation of *CDK5R1* expression, including an important class of post-transcriptional regulators, the microRNAs (miRNAs). miRNAs are short, on average only 22 nucleotides long, non-coding RNAs whose action usually results in mRNA degradation or translation repression, depending on the degree of sequence complementarity with the 3′-UTR of their target transcripts. Animal miRNAs typically have imperfect complementarity with their target mRNAs [29], and this causes translational repression. Target prediction algorithms have estimated that thousands of human gene products are regulated by miRNAs [30]. Current functional studies have shown that miRNAs are key regulators of developmental processes, such as self-renewal of stem cells, myogenesis, embryogenesis, and cellular differentiation [31]–[33]. Many miRNAs are expressed in the CNS, often in a temporally and/or spatially regulated manner during development, differentiation and neuronal survival, and are also potentially involved in neuronal plasticity and learning with a reported role in neurodegeneration [34], [35].

Given the functional necessity of maintaining a correct cellular level of p35, miRNAs are also expected to be involved in the fine tuning of p35 expression. In the present study we report on the identification of two miRNAs, miR-103 and miR-107, regulating *CDK5R1*/p35 expression and reducing migration of the neuroblastoma cells SK-N-BE. Among the several predicted miRNA binding sites, we validated the activity of one miR-107 and one miR-103 binding site by means of functional studies based on luciferase assays.

## Results

### Prediction of miRNA target sites in *CDK5R1* 3′-UTR

In order to identify miRNAs potentially regulating *CDK5R1* expression, we first searched for miRNAs predicted to target the *CDK5R1* 3′-UTR, using the algorithm PicTar. We found that the 3′-UTR of human *CDK5R1* harbours putative target sites for 20 different miRNAs (Table 1). The number of predicted binding sites for each miRNA varies from one to 13. Moreover, each target site can be bound by a number ranging from 1 to 8 different miRNAs. Because the regulatory effects of miRNAs are typically seen to increase when more than one molecule of the same miRNA binds its targets [36], we decided to select for subsequent study the six miRNAs (miR-195, miR-16, miR-15a, miR-15b, miR-107 and miR-103) which were predicted to bind to the highest number of target sites (≥10).

**Table 1:** PicTar miRNA target sites prediction in *CDK5R1* 3′-UTR.

microRNA	# of sites	Free energies (kcal/mol)
has-miR-15b	13 (2) (5)	−19.2 −22.5 −17.6 −18.6 −16.8 −18.2 −19.7 −18.1 −16.3 −17.1 −22.5 −16.1 −16.0
hsa-miR-195	12 (2) (4)	−16.9 −17.5 −18.6 −18.7 −17.3 −18.6 −20.6 −17.6 −21.1 −16.4 −17.4 −19.0
has-miR-107	11 (7) (3)	−25.3 −21.2 −22.2 −19.9 −19.4 −19.4 −24.9 −24.3 −23.5 −24.5 −16.8
has-miR-15a	11 (6) (3)	−20.9 −21.0 −20.3 −16.8 −19.4 −23.1 −20.1 −18.6 −19.0 −24.7 −17.9
has-miR-103	10 (7) (2)	−25.3 −21.2 −22.2 −19.4 −18.2 −23.5 −23.9 −24.5 −24.2 −16.8
has-miR-16	10 (2) (5)	−19.3 −19.1 −18.4 −15.1 −19.5 −23.2 −18.9 −22.8 −17.3 −16.3
has-miR-130b	6 (4) (0)	−16.1 −21.5 −24.8 −15.9 −21.9 −24.6
has-miR-130a	6 (2) (1)	−16.1 −22.1 −16.4 −15.4 −19.1 −22.4
has-miR-33	6 (1) (1)	−16.9 −18.3 −20.0 −11.3 −17.3 −17.3
has-miR-183	4 (4) (0)	−20.3 −23.2 −20.1 −20.5
has-miR-27b	4 (3) (1)	−20.7 −22.6 −18.2 −22.8
has-miR-23a	4 (2) (1)	−24.4 −18.7 −24.5 −13.8
has-miR-23b	4 (2) (1)	−22.4 −18.2 −23.1 −13.8
has-miR-25	4 (1) (0)	−17.2 −22.5 −15.4 −17.9
has-miR-301	3 (2) (0)	−21.1 −15.8 −22.0
has-miR-27a	3 (2) (0)	−20.7 −17.6 −20.0
has-miR-152	3 (2) (0)	−25.0 −15.6 −20.7
has-miR-148a	2 (2) (0)	−20.0 −21.4
has-miR-148b	2 (2) (0)	−20.7 −23.6
has-miR-101	1 (0) (1)	−19.4

In the second column, the numbers are: number of binding sites for the given miRNA in *CDK5R1* 3′-UTR, number of binding sites with a free energy smaller that −20.0 kcal/mol., number of binding sites with free energy between −20.0 and −18.0 kcal/mol.

### Inverse correlation between miR-103 and miR-107 expression and p35 level in human cell lines

**Figure 1:**
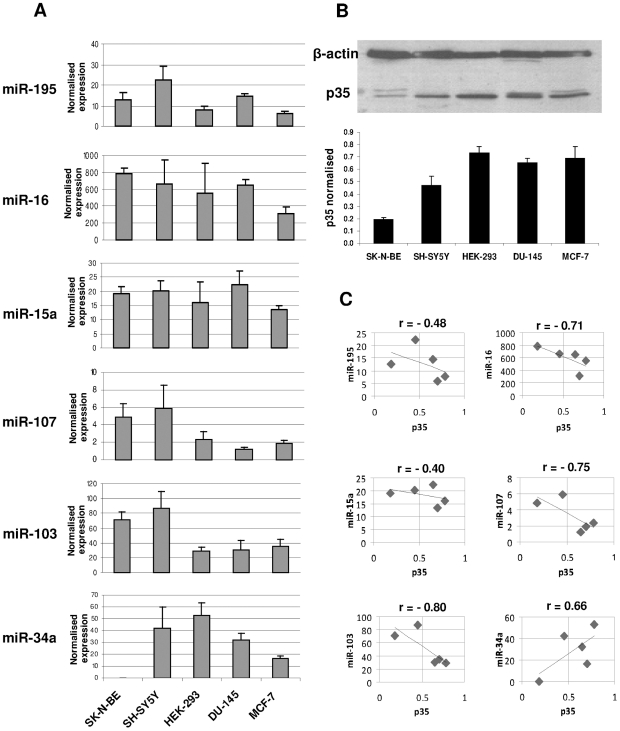
Correlation between p35 and miRNAs expression in cell lines. **A**) Expression of mature miR-195, miR-16, miR-15a, miR-107, miR-103 and miR-34a measured by Real-Time PCR on total RNA extracted from SK-N-BE, SH-SY5Y, HEK-293, DU-145 and MCF-7 cell lines. The expression levels are normalised on the expression of the endogenous control RNU6B. RNU6B levels were comparable in all samples tested. **B**) Western blot for p35 detection in protein extracts from the same cell lines. Expression is normalised on β-actin. A representative experiment is shown at the top of the panel. **C**) Correlation analysis between each miRNA and p35 protein levels in the five cell lines, r = Pearson's correlation coefficient.

Preliminary RT-PCR experiments on miRNA precursors (pre-miRs) in five neuronal and non neuronal human cell lines (SK-N-BE, SH-SY5Y, HEK-293, DU-145, MCF-7) showed the expression of all but one (miR-15b) of the miRNAs in all of the tested cell lines (data not shown). We then sought to identify which among the five expressed miRNAs could be the most plausible candidates for having a regulatory effect on *CDK5R1* expression by evaluating the expression levels of the mature miRNAs by qRT-PCR in the above mentioned human cell lines (Figure 1A), and comparing them with the levels of p35, encoded by *CDK5R1*, as quantified by Western blot (Figure 1B). miR-34a was included in this analysis as a negative control, given that it was not predicted to target *CDK5R1* mRNA. Neuronal cell lines (SK-N-BE and SH-SY5Y) showed significantly lower amounts of p35 compared to the non neuronal cell lines (HEK-293, DU-145 and MCF-7) (Figure 1B). Each miRNA displays a particular expression profile in the studied cell lines (Figure 1A). Since post-transcriptional miRNA-mediated regulation usually results in gene silencing, we expect an inverse correlation between miRNA and p35 levels in the five cell lines only for those miRNAs which have a regulatory action on *CDK5R1* expression. We then performed a correlation analysis, based on Pearson's correlation coefficient, which revealed that there is a negative correlation for all the five miRNAs, with correlation coefficients ranging from −0.40 to −0.80 (Figure 1C). Conversely, miR-34a did not show a negative correlation coefficient (r = 0.66), as expected for a miRNA without predicted targets sites in *CDK5R1* 3′-UTR. Out of the three miRNAs with the most negative correlation coefficients (miR-103: r = −0.80; miR-107: r = −0.75; miR-16: r = −0.71), we decided to focus our attention on miR-103 and miR-107 as potential modulators of *CDK5R1* expression by virtue of the lowest free energy values of their predicted binding sites (Table 1). Of note, miR-103 and miR-107 have the same sequence with the exception of just one nucleotide located outside the so-called ‘seed sequence’ and, by consequence, share all but one of the predicted target sites.

### miR-103 and miR-107 modulate *CDK5R1* expression in SK-N-BE cells

To assess the effects of miR-103 and miR-107 on endogenous *CDK5R1* expression, we transiently transfected SK-N-BE cells with either their precursors (pre-miR-103 and pre-miR-107) or their inhibitors (anti-miR-103 and anti-miR-107). qRT-PCR on mature miRNAs confirmed that the transfection of the respective miRNA precursors lead to miR-103 and miR-107 overexpression, compared to the untransfected controls. Vice versa, the transfection of miRNA inhibitors almost abolished the expression of miR-103 and miR-107 (data not shown).

**Figure 2:**
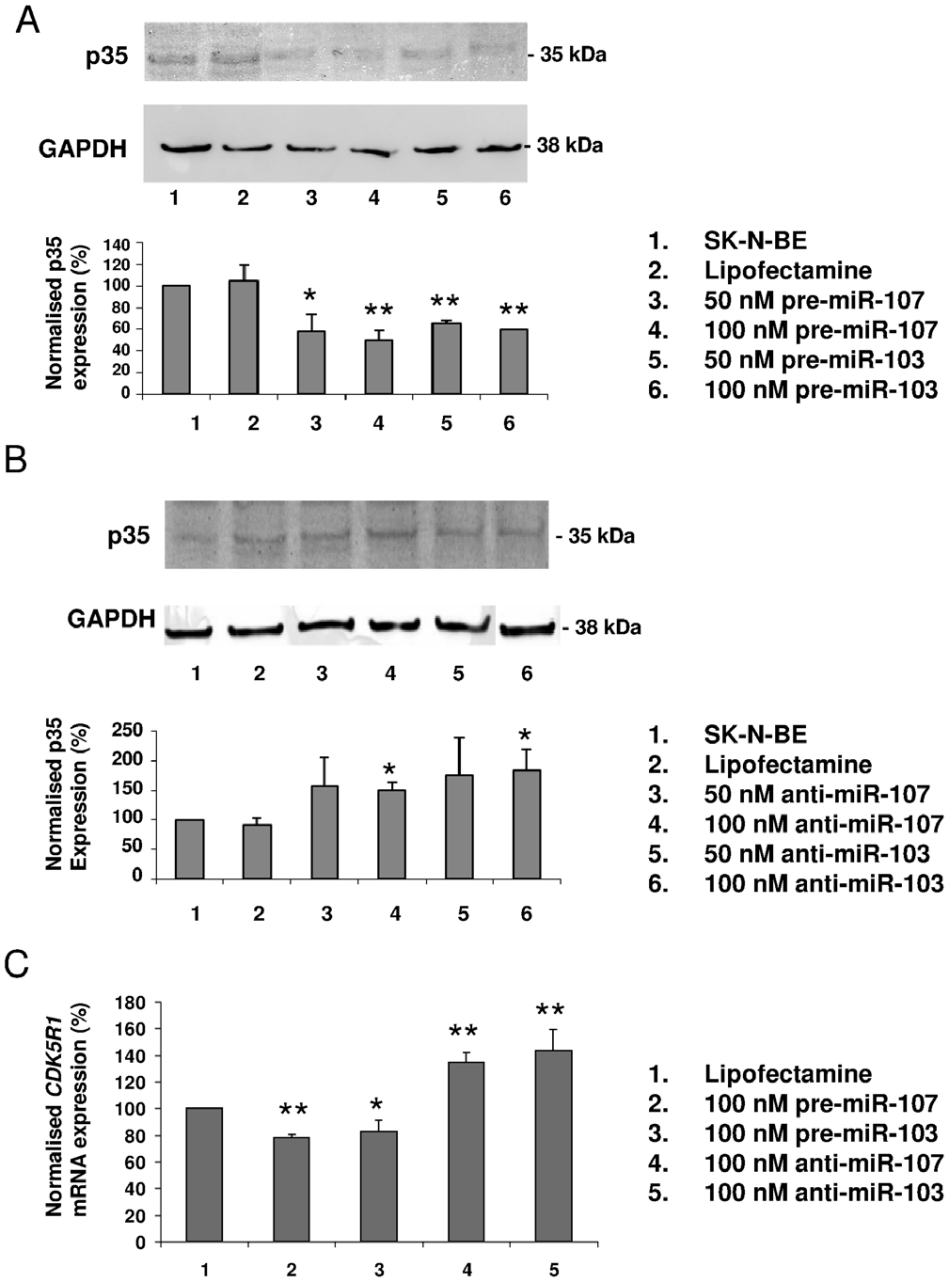
p35 protein and *CDK5R1* mRNA expression in pre-miR-103/107 and anti-miR-103/107 transfected and untransfected SK-N-BE cells. **A**) Western blot analysis of p35 in the conditioned media of control SK-N-BE cells (lane 1 and 2), 50 nM and 100 nM pre-miR-103 and pre-miR-107 transfected cells, at 48 h from transfection. A representative experiment is shown at the top of the panel. (*p<0.05, **p<0.01). **B**) Western blot analysis of p35 in the conditioned media of control SK-N-BE cells (lane 1 and 2), 50 nM and 100 nM anti-miR-103 and anti-miR-107 transfected cells, at 48 h from transfection. A representative experiment is shown at the top of the panel. (*p<0.05). **C**) *CDK5R1* mRNA expression level measured by qRT-PCR after 48 h from transfection with 100 nM pre-miR-107, pre-miR-103, anti-miR-107 and anti-miR-103 normalised on housekeeping *GAPDH* transcript. The level of *GAPDH* expression was comparable in all samples tested. (*p<0.05, **p<0.01).

48 h after pre-miR-107 transfection, a significant reduction of p35 levels was observed, by 42% with 50 nM and 51% with 100 nM of precursor, compared to the untransfected control after normalization on GAPDH levels. Similarly, the transfection of pre-miR-103 caused a 35% (50 nM) and a 40% (100 nM) reduction of p35 levels (Figure 2A). Otherwise, if anti-miR-107 (50–100 nM) was transfected, an increase of 57% and 50% in p35 levels was observed, respectively, compared to the untransfected control. Moreover, an increase of 76% (50 nM) and 83% (100 nM) of p35 levels was observed following anti-miR-103 transfection (Figure 2B).

Total mRNA was extracted from SK-N-BE cells 48 hours after transfection with 100 nM pre-miR-103, pre-miR-107, anti-miR-103 and anti-miR-107. A qRT-PCR showed a decrease in *CDK5R1* transcript level after transfection with the miRNA precursors and an increase of *CDK5R1* mRNA after treatment with the anti-miRNAs (Figure 2C). Taken together, these results suggest that miR-103 and miR-107 can affect *CDK5R1* expression in SK-N-BE cells.

### miR-107 directly regulates *CDK5R1* translation

**Figure 3:**
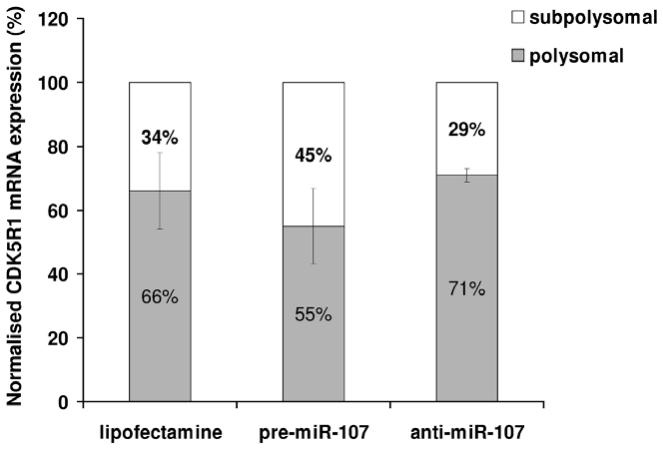
Effect of miR-107 on *CDK5R1* mRNA in polysomal and subpolysomal lysates. qRT-PCR of *CDK5R1* mRNA in polysomal and subpolysomal fractions. SK-N-BE cells were transfected with 100 nM pre-miR-107 or anti-miR-107 for 48 h, then the mRNA–ribosomal complexes were separated through sucrose gradient centrifugation. Expression is normalized on the housekeeping *Alu* transcript. The percentages of the transcript obtained from the two fractions are calculated on the sum of the 2^−ΔCt^ of the mRNA from polysomal and subpolysomal compartments, for pre-miR-107, anti-miR-107 treatments and the control (lipofectamine).

To verify whether miR-107 directly regulates the level of p35 by affecting translation efficiency of *CDK5R1* mRNA, the SK-N-BE cell line was transfected with pre-miR-107 or anti-miR-107, then after 48 h the mRNA–ribosomal complexes were separated through sucrose gradient centrifugation in the polysomal and subpolysomal fractions, containing the efficiently translated transcripts and the untranslated mRNAs respectively. After RNA extraction a qRT-PCR has been performed to quantify *CDK5R1* mRNA, allowing us to determine, on the basis of the ΔCt values, the amount of this transcript in both polysomal and subpolysomal compartments. The relative distributions of *CDK5R1* mRNA in the two compartments are indicated as percentages calculated on the sum of the transcript extracted from the two fractions, for each treatment and control (Figure 3). The results, despite not reaching the statistical significance, show a trend towards a shift of *CDK5R1* mRNA from the polysomal to the subpolysomal compartment after transfection with pre-miR-107 compared to the control (Figure 3), indicating that miR-107 directly acts as a negative translational regulator. On the other hand, transfection with anti-miR-107 suggest a shift of the mRNA from the subpolysomal to the polysomal fraction compared to the control (Figure 3), indicating an increase in translation. These results confirm that miR-107 directly regulates CDK5R1 expression at translational level.

### miR-103 and miR-107 interact with the *CDK5R1* 3′-UTR

**Figure 4:**
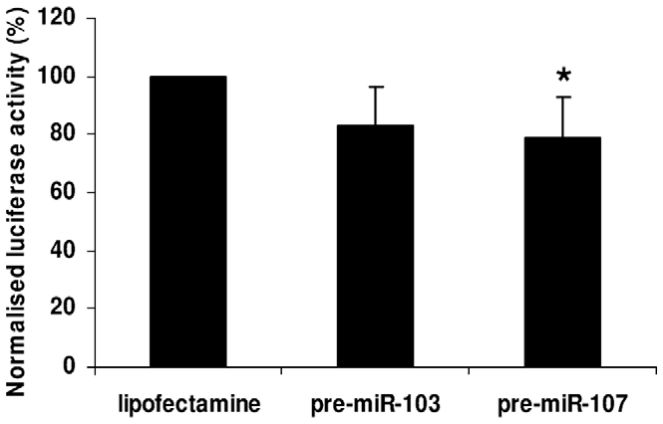
Interaction between miR-103/107 and *CDK5R1* 3′-UTR. The 2688 bp sequence of the *CDK5R1* 3′-UTR was cloned upstream the Renilla luciferase gene generating the pGL4.71P-UTR construct. This was cotransfected into SK-N-BE cells with a firefly luciferase report vector for luciferase normalization. Cells were treated with the transfection agent alone (Lipofectamine) or with 100 nM pre-miR-103 or pre-miR-107. Luciferase activity was assessed 48 h after transfection of pre-miRNAs (*p<0.01).

To verify that miR-103 and miR-107 act on p35 levels by directly interacting with the *CDK5R1* 3′-UTR, we co-transfected into SK-N-BE cells the pGL4.71P-UTR construct, containing the whole human *CDK5R1* 3′-UTR downstream of the *Renilla* luciferase coding sequence [23], with 100 nM of pre-miR-103 or pre-miR-107. The co-transfection in SK-N-BE of this construct with pre-miR-103 or pre-miR-107 led to a reduction of luciferase activity (Figure 4), in comparison to the luciferase activity measured in the same cells transfected only with pGL4.71P-UTR, suggesting a direct action of the two miRNAs on *CDK5R1* transcript by means of interaction with its 3′-UTR.

**Figure 5:**
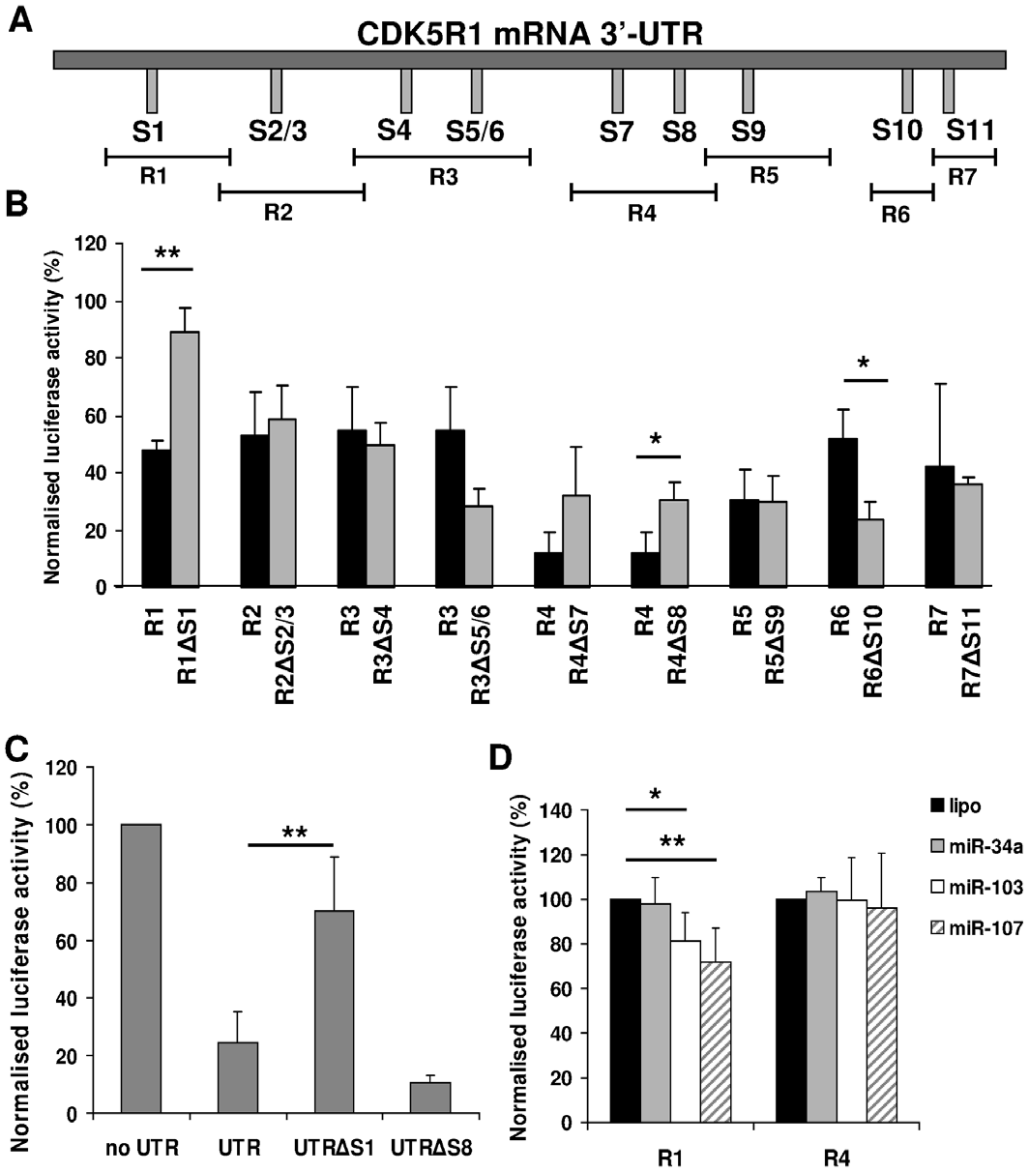
Functional analysis of miR-103/107 target sites on *CDK5R1* 3′-UTR. **A**) Schematic location of miRNA target sites (S1 to S11) in the *CDK5R1* 3′-UTR and schematic representation of the regions of 3′-UTR (named from R1 to R7) used to generate the luciferase constructs. **B**) SK-N-BE cells were transfected with the *CDK5R1* 3′-UTR-luciferase constructs, each one containing one or two predicted binding sites or with the same constructs deleted of the binding sites (named ΔS constructs). 24 h after transfection, cells were collected and then Renilla luciferase activities were estimated and normalized to firefly luciferase activities. The deletion of both S1 and S8 lead to an increase in luciferase activity compared to the wild-type construct. (*p<0.05, **p<0.01). **C**) SK-N-BE cells were transfected with the pGL4.71P control plasmid (no UTR), with pGL4.71P-UTR either wild-type (UTR) or deleted of S1 (UTRΔS1) or S8 (UTRΔS8). Deletion of S1 leads to an increase of luciferase activity compared to the wild-type UTR. (**p<0.01). **D**) Luciferase constructs R1, containing S1, and R4, containing S8, have been transiently co-transfected in SK-N-BE cells and separately treated with 100 nM pre-miR-103 and pre-miR-107. Pre-miR-34a was used as a control as it can not bind *CDK5R1* 3′-UTR. Luciferase activity of the R1 construct is significantly diminished when transfected with pre-miR-103 or pre-miR-107. (*p<0.05, **p<0.01).

We then attempted to establish which among the different miR-103 and miR-107 predicted target sites were the most effective in modulating *CDK5R1* expression. The bioinformatic analysis of the 3′-UTR predicted the presence of 10 and 11 target sites (S1–S11) for miR-103 and miR-107, respectively. Indeed, the S5 site is recognised by PicTar as a target site for miR-107 but not for miR-103. Moreover, since S2 and S3 sites, as well as S5 and S6 sites, are mostly overlapped, we treated them as single sites, referred as to site S2/3 and S5/6, respectively. We divided the 3′-UTR into 7 regions (named R1 to R7), each of them containing one or two binding sites (Figure 5A). Following the cloning of each fragment in the pGL4.71P plasmid and the generation of the respective constructs in which the seed region of each site was deleted, we transfected the wild-type and the deleted constructs in SK-N-BE cells, to investigate the activity of endogenous miR-103 and miR-107 on each predicted binding site (Figure 5B). Luciferase assays showed that the reporter gene activity of all the wild-type constructs was reduced with respect to the control pGL4.71P plasmid, which has been set to 100. Comparison between each wild-type fragment and the respective construct deleted of the seed region showed a significant increase of luciferase activity following deletion of S1 and S8, but not the remaining binding sites, suggesting their contribution to the down-modulation of the reporter gene (Figure 5B). S1 and S8 were also deleted from the pGL4.71P-UTR in order to study their effect on the whole 3′-UTR (Figure 5C). Only the deletion of S1 led to a significant increase in luciferase activity.

In order to study whether miR-103 and miR-107 display activity on S1 and S8 sites, the constructs R1, containing S1, and R4, containing S8, have been transiently transfected and treated with pre-miR-103 and pre-miR-107 in SK-N-BE cells. Pre-miR-34a was used as a negative control as it does not target *CDK5R1* 3′-UTR. As shown in Figure 5D, luciferase activity of the R1 construct is significantly diminished when transfected with miR-103 or miR-107, while R4 construct, is not affected following transfection with the two miRNAs, indicating that this site is not sensitive to miR-103 and miR-107 exposition. Also, the transfection of miR-34a does not lead to any change in luciferase activity, as expected. These data suggest that miR-103 and miR-107 display an effect on *CDK5R1* regulation through the binding to the S1 site. It is worth to be noted that the S1 site shows the lowest hybridization free energy with miR-103 and miR-107, based on both PicTar (Table 1) and RNAhybrid analysis (Figure S1 and S2).

### miR-103, miR-107 and *CDK5R1* silencing reduce the migration ability of SK-N-BE cells

**Figure 6:**
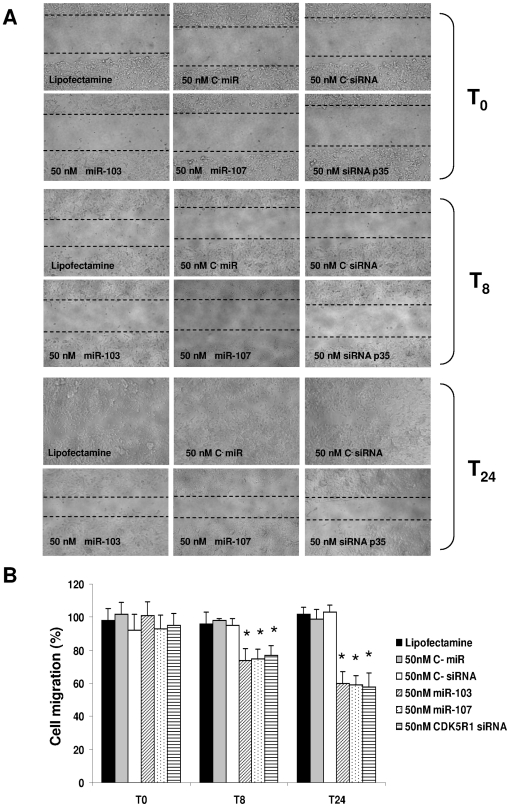
Measurement of cell migration by *in vitro* scratch assay. SK-N-BE cells were transiently transfected with 50 nM pre-miR-103, pre-miR-107 or control miR (C- miR) and with 50 nM CDK5R1 siRNA or control siRNA (C- siRNA). The migration ability was assessed 8 h and 24 h from the scratch. **A**) The figure shows the cells immediately after the scratch (T_0_), 8 h (T_8_) and 24 h (T_24_) after the scratch. Magnification is 10×. **B**) After 8 h (T_8_) from the scratch, the migration was inhibited by 25% in 50 nM pre-miR-107 transfected cells, by 26% in 50 nM pre-miR-103 transfected cells and by 23% in 50 nM *CDK5R1* siRNA transfected cells, compared to untransfected cells. After 24 h (T_24_) from the scratch, the migration was inhibited by 41% in 50 nM pre-miR-107 SK-N-BE transfected cells, by 40% in 50 nM pre-miR-103 transfected cells and by 42% in 50 nM *CDK5R1* siRNA transfected cells. (*p<0.01).

Given the fundamental role of p35 in neuronal migration and the evidence that miR-103 and miR-107 can modulate endogenous p35 levels, we examined whether their transfection also affected SK-N-BE cell migration capacity. As shown in Figure 6, 8 hours after transfection the migration abilities of the 50 nM pre-miR-103 and pre-miR-107 transfected cells were significantly reduced up to 31% and 28% respectively; 24 hours after transfection the migration abilities of the 50 nM pre-miR-103 and pre-miR-107 transfected cells were significantly reduced up to 42% and 46%. No significant effects on cell migration capacity was seen after transfection of 50 nM control miRNA. The inhibition percentages are calculated setting the SK-N-BE migration ability at 100%. To support the assertion that miR-103 and miR-107 affected the SK-N-BE migration through the regulation of the p35 level, we knocked-down p35 by *CDK5R1* siRNA transfection in SK-N-BE cells. The p35 protein levels were decreased up to 41% at 48 hours (Figure S3, panel A) and 42% at 72 hours (Figure S3 panel B) after 50 nM and 100 nM *CDK5R1* siRNA cell transfection. The p35-knocked-down cells were subsequently monitored for their migration ability by means of a scratch assay. 8 hours after transfection the migration abilities of 50 nM *CDK5R1* siRNA transfected cells were significantly reduced up to 23% and up to 44% at 24 hours from transfection. The transfection of 50 nM control siRNA did not affect cellular migration. The scratch assay was also performed transfecting 100 nM of each molecule, leading to comparable results (data not shown). These results suggest that miR-103 and miR-107 are involved in cell migration by p35 inhibition.

## Discussion

p35, encoded by *CDK5R1*, is a short-lived protein and the fine regulation of its level in the cell is crucial for CDK5 activity [21]. The regulation of p35 stability by PKCδ has been recently reported [22] and the regulatory action of *CDK5R1* 3′-UTR on the post-transcriptional control of its expression has also been investigated [23], even if the influence on *CDK5R1*/p35 levels by miRNAs, an important class of post-transcriptional regulatory factors, has never been investigated.

miRNAs have been implicated in several cellular processes such as proliferation, differentiation and apoptosis and, taking into account the high complexity of the mammalian brain, miRNAs emerge as important factors in the regulation of specific functions in CNS such as neuronal migration, differentiation and synaptic plasticity [37].

Here we have shown that miR-103 and miR-107 affect p35 expression in SK-N-BE neuroblastoma cells and their overexpression reduces cellular migration. Functional studies demonstrate the direct action of the two miRNAs on the *CDK5R1* 3′-UTR, allowing us to identify a functional binding site.

Analysis of the *CDK5R1* 3′-UTR by PicTar allowed to select six miRNAs for the presence of more than 10 binding sites within *CDK5R1* 3′-UTR, interestingly belonging to the same family, known as the miR-15/107 family [38]. These miRNAs present an AGCAGC motif near the critical 5′ end of the mature miRNA and are known to regulate the expression of genes involved in cell division, metabolism, stress response and angiogenesis. The miR-15/107 group has also been implicated in human cancers, cardiovascular disease and neurodegenerative disease [38].

Among the selected miRNAs, miR-103 and miR-107 are those whose expression levels showed the most negative Pearson's correlation coefficient when compared with p35 level. Besides they are also predicted to bind seven target sites with a free energy <−20 kcal/mol (Figure 1, Table 1). miR-103 and miR-107 differ in their mature sequence for only one base, thus are referred to as paralogous miRNAs and are predicted to bind the same target sites, except for one, in the *CDK5R1* 3′-UTR. miR-103 is encoded by two different genes, *MIR103-1* (GeneID: 406895) and *MIR103-2* (GeneID: 406896), residing within the *PANK3* (GeneID: 79646; chromosome 5q34) and *PANK2* (GeneID: 80025; chromosome 20p13) genes, respectively, which give rise to an identical mature miRNA. Instead, the primary transcript of miR-107 (*MIR107* GeneID: 406901) is co-expressed with an intronic segment of *PANK1* (GeneID: 53354), a coding gene localized on chromosome 10. miR-103 and miR-107 genes are completely conserved in all known vertebrates. They have been shown to be expressed in many human organs with the highest concentration in brain tissue, in particular they are abundantly expressed in upper cortical layers and in white matter, with a laminar specificity in human prefrontal cortex [39], [40]. The two miRNAs are differentially expressed in a number of contexts, including development [41], [42], oncogenesis [43], neurodegeneration [38], hypoxia [44], and both cold and heat stress [45], [46]. Only a few targets of miR-103 and miR-107 have currently been validated: *beta-site amyloid precursor protein-cleaving enzyme 1* (*BACE1*, GeneID: 933) [47], *cyclin-dependent kinase 6* (*CDK6*, GeneID: 1777) [48], *granulin* (*GRN*, GeneID: 4601) [49] and *DICER1* (*DRCE1*, GeneID: 17098) [50], while the *brain-derived neurotrophic factor* (*BDNF*, GeneID: 1033) is indicated as a potential target [51].

The effect of miR-103 and miR-107 on p35 expression is consistent with their direct action on both *CDK5R1* mRNA degradation, as observed by qRT-PCR on total RNA, and on *CDK5R1* mRNA translation efficiency, as established following qRT-PCR on polysomal and subpolysomal fractions. As BDNF is known to stabilize p35 and its transcript is also a possible target of miR-103 and miR-107 [22], [51], we cannot exclude that the effect on p35 expression may be enhanced by BDNF action, since p35 and BDNF are both regulated by the two studied miRNAs. p35 downregulation by overexpression of miR-103 and/or miR-107 as well as by *CDK5R1* silencing lead to reduced migration ability of SK-N-BE cells. These findings suggest that the effect of miR-103 and/or miR-107 on cellular migration acts through p35 downregulation. This proposed mechanism is in accordance with neuronal migration impairment in *Cdk5r1* KO mice, inferred by the observation of an inverted cortex lamination [11]. This evidence indicates that the two miRNAs can play a role in the regulation of cellular migration, a function so far proved only for a few miRNAs [52].

In accord, miR-103 and miR-107 can be included in the group of seven miRNAs (miR-30a-5p, miR-30b,c,d, miR-103/107, miR-191, miR-195) possibly involved in neuronal migration, through the regulation of *BDNF* expression in human cortex [51]. Interestingly, BDNF activates PKCδ which in turn stabilizes p35, increasing CDK5 activation. PKCδ was demonstrated to regulate the radial migration of layer II/III cortical neurons and both PKCδ and p35 are required for BDNF-stimulated migration in the culture of cortical newborn neurons [22]. Furthermore, the correct neuronal migration and cortical lamination strongly depends on CDK5 activation by p35, through the regulation of motor proteins and cytoskeleton components engaged in the cell motility [53]. It is known that p35 protein undergoes fast turnover in an ubiquitin- and proteasome-dependent manner [21] and its level is rate-limiting for CDK5 activity [17]. Thus, the maintenance of proper CDK5 activity in migrating neurons strongly depends on the level of p35 which therefore needs to be precisely regulated. *Cdk5r1* KO mice show inverted stratification of cerebral cortex layers due to an abnormal neuronal migration, probably impairing the development of a normal cognitive system. *CDK5R1* haploinsufficiency has been invoked as one of the possible pathogenetic causes of mental retardation in NF1 microdeletion patients [16] and some variants of this gene have been associated with isolated mental retardation [54].

The importance of maintaining suitable p35 levels probably involves different molecular mechanisms aimed at the fine tuning of its expression through protein stabilization and by regulation of mRNA stability or translational efficiency by means of the action of post-transcriptional regulatory factors such as miRNAs. Dysregulation of miRNAs has also been reported in neurological and neurodegenerative disorders, which leaves open the question of whether miRNA dysfunction could be both a cause and/or a consequence of disease [55].

One of the main CDK5 functions is phosphorylation of proteins which stabilize microtubules, such as Tau [56]. Abnormal hyperphosphorylation of Tau, also depending on CDK5 hyperactivation, appears to be the most deleterious step in neurofibrillary degeneration, leading to the formation of neurofibrillary tangles which are associated with Alzheimer's disease [57]. Interestingly, miR-107 has been shown to be significantly down-regulated in Alzheimer's disease and one of its targets, *BACE1*, was found to be increased [47]. Accordingly, an increased level of p35 might be caused by an increase of protein stabilization by BDNF and/or by an up-regulation following a down-regulation of miR-107, leading to hyperactivation of CDK5 in Alzheimer's disease. Intriguingly, the implication of miR-107 in the regulation of p35 expression indicates that *BDNF* and *BACE1* may be co-regulated with *CDK5R1* in the CNS.

The direct interaction of miR-103 and miR-107 with the *CDK5R1* 3′-UTR has been proved by functional studies using luciferase constructs with the whole *CDK5R1* 3′-UTR, or with a portion of the 3′-UTR including one, or in a few cases two, binding sites. The increased luciferase activity in constructs deleted of S1 and S8 binding sites suggests their destabilizing function. Unexpectedly we observed that the deletion of S5/6 and S10 sites decreased the luciferase activity of R3 and R6 constructs, respectively. Even if we verified that the mutagenesis did not create miRNA target sites or recognizable *cis*-regulatory elements by using different bioinformatics tools, we can not exclude that unknown *cis*-regulatory elements or miRNA target sites or secondary structures destabilizing mRNA may have been created by our manipulation. The co-transfection of constructs containing S1 or S8 sites with miR-103 or miR-107 allowed us to validate the direct action of the two miRNAs for the S1 target site. This result can be explained by the lower hybridization free energy with miR-103 and miR-107 of S1, which has the lowest hybridization free energy of all the predicted miRNA target sites, based on both PicTar and RNAhybrid tools. We have to take into account that the luciferase activity of all the wild-type constructs was reduced with respect to the control pGL4.71P plasmid, due to the presence of several post-transcriptional regulatory elements with a inhibitory effect on gene expression all along *CDK5R1* 3′-UTR [23]. This feature made the detection of further decrease of luciferase activity more difficult. In addition, it is worth noting that S1 is specific for miR-103 and miR-107, while S8 can also be targeted by miR-15a and miR-195, whose action may explain the increase of luciferase activity in the construct deleted of S8 binding site.

The conservation of the *CDK5R1* 3′-UTR sequence during evolution, the identification of different regulatory regions [23] and the prediction of several miRNA binding sites with a low binding free energy pinpoint the functional complexity of this region in post-transcriptional regulation. We have here demonstrated that *CDK5R1*/p35 expression can be modulated by miR-103 and miR-107, which we found to regulate cellular migration, accordingly with the roles ascribed to their targets BDNF and p35 in previous studies [11], [51]. Knowing that the level of CDK5 activity depends on the amount of p35, we speculate that p35 level can be finely controlled by both a protein stabilization mechanism involving BDNF, and by a post-transcriptional expression modulation involving miR-103 and miR-107, which in turn co-regulate the level of p35 and its stabilizing factor BDNF.

Studies providing new insights on the identification of miRNAs targets, may lead to the elucidation of specific molecular pathways, also defining their implication in specific cellular functions. The identification of regulatory sites in *CDK5R1* mRNA and their binding factors, besides contributing to the understanding of the role of the post-transcriptional regulation of this gene, may address studies aimed at identifying new pathogenetic mechanisms in neuronal diseases.

## Materials and Methods

### miRNA target sites prediction

The prediction of miRNA target sites was performed using the algorithm PicTar (http://pictar.mdc-berlin.de/). The analysis of the hybridization free energy between the miRNAs and the predicted target sites was performed using RNAhybrid (http://bibiserv.techfak.uni-bielefeld.de/rnahybrid/).

### Cell cultures

Human neuroblastoma SK-N-BE (2) cells (ATTC code CRL-2271) were cultured in RPMI-1640 medium with 10% fetal bovine serum (FBS), 100 U/ml penicillin-streptomycin, 0.01 mM L-glutamine, sodium pyruvate 11 g/l and glucose 4.5 g/l. Human neuroblastoma SH-SY5Y cells (ATTC code CRL-2266) and human embryonic kidney HEK-293 cells (ATTC code CRL-1573) were maintained in DMEM medium with 10% FBS, 100 U/ml penicillin-streptomycin and 0.01 mM L-glutamine. Human prostate carcinoma DU-145 cells (ATTC code HTB-81) were cultured in RPMI-1640 medium with 10% FCS, 100 U/ml penicillin-streptomycin, 0.01 mM L-glutamine and 10 mM HEPES. Human breast tumor MCF-7 cells (ATTC code HTB-22) were cultured in RPMI-1640 medium with 10% FCS, 100 U/ml penicillin streptomycin and 0.01 mM L-glutamine (all media ingredients were obtained from Euroclone, Italy). Cultures were maintained at 37°C in a 5% CO2 incubator.

### Western blotting

The assay was performed under standard conditions. In brief, equal amounts of proteins were analyzed by 12% SDS-PAGE, blotted onto nitrocellulose membrane Hybond-enhanced chemiluminescence (ECL; Amersham Biosciences UK, Ltd., Buckinghamshire, UK) in a Bio-Rad (CA, USA) Trans-blot apparatus at 100 V for 90 min. Blots were processed by an ECL Plus detection kit as instructed by the supplier (Amersham Biosciences). The blots were probed with rabbit anti-p35 (C-19) polyclonal antibody (Santa Cruz Biotechnology, Inc., CA, USA) and rabbit anti-β-actin (Sigma-Aldrich, CO, USA) or anti GAPDH antibodies followed by an alkaline phosphatase-conjugated anti-rabbit IgG secondary antibody. All experiments were performed three times.

### Pre-miRs, anti-miRs and siRNA transfection

dsRNAs that mimic endogenous mature miRNAs (hsa-miR-103: 5′- AGCAGCAUUGUACAGGGCUAUGA-3′ and hsa-miR-107: 5′- AGCAGCAUUGUACAGGGCUAUCA-3′), the Pre-miR™ miRNA Precursor Molecules—Negative Control #1 and single-strand anti-miR-103/107 miR inhibitor (oligoribonucleotides), designed to inhibit endogenous hsa-miR-103/107, were purchased from Ambion (Austin, TX, USA). siRNA ON TARGET PLUS smart pool CDK5R1 or non-targeting pool were purchased from Dharmacon (Lafayette, CO, USA). The transfection experiments were conducted as described by Salvi [58]. Briefly, SK-N-BE cells were seeded in complete medium at 80% confluence in a 24-well Costar (5×10^4^ cells per well). The cells were transfected into serum-free RPMI, 24 h after seeding, at 50 and 100 nM of double-stranded hsa-miR-103/107 or single-stranded anti-miR-103/107 miRNA inhibitor or with 50 nM and 100 nM *CDK5R1* siRNA, using Lipofectamine 2000 (Invitrogen, Burlington, ON) transfection reagent according to the manufacturer's instruction. The transfection had a very high efficiency as proved by measuring of mature miRNA molecules levels by qRT-PCR after transfection (data not shown). The transfection medium was replaced, after 24 h, with RPMI supplemented with 10% fetal bovine serum. The conditioned media and cell extracts were prepared for analysis 48 and 72 h after the transfection.

### Polysomal RNA extraction

SK-N-BE cells (4×10^6^), transfected as described above, were incubated for 3 minutes with 0.01 mg/ml cycloheximide at 37°C then the plate was put on ice. The media was removed and the cells were washed twice with cold phosphate buffer saline (PBS) + cycloheximide 0.01 mg/ml. Cells were directly lysed on the plate with 300 µl cold lysis buffer [10 mM NaCl, 10 mM MgCl_2_, 10 mM Tris–HCl, pH 7.5, 1% Triton X-100, 1% sodium deoxycholate, 0.2 U/µl RNase inhibitor (Fermentas, Burlington, CA), 1 mM dithiothreitol and 0.01 mg/ml cycloheximide], scraped and transferred to an Eppendorf tube. The extracts were centrifuged for 5 min at 12000 g at 4°C. The supernatant was frozen in liquid nitrogen and stored at −80°C or loaded directly onto a 15–50% linear sucrose gradient containing 30 mM Tris–HCl, pH 7.5, 100 mM NaCl, 10 mM MgCl_2_, and centrifuged in an SW41 rotor for 100 min at 180000 *g*. Fractions (polysomal and sub-polysomal) were collected monitoring the absorbance at 254 nm and treated directly with 0.1 mg/ml proteinase K for 2 hours at 37°C. After phenol–chloroform extraction and isopropanol precipitation, polysomal and sub-polysomal RNAs were resuspended in 30 µl of RNAse free water and then repurified with RNeasy kit (Qiagen, Hilden, Germany).

### Real-time PCR

The total RNAs from transfected and nontransfected cells were isolated using TRIzol reagent (Invitrogen), according to the manufacturer's instructions. Purity of RNAs (A260/A280 value of 1.8–2.1) and concentration was measured using Nanodrop spectrophotometer.

For a quantitative analysis of mature miRs, a two-step Taq-Man real-time PCR analysis was performed, using primers and probes obtained from Applied Biosystems (Foster, CA, USA). cDNA was synthesized from total RNA (50 ng) in 15 µl reactions, using reverse transcriptase and the stem–loop primer for miR-15a (ID000389), miR-16 (ID000391), miR-103 (ID000439), miR-107 (ID000443), miR-195 (ID000494), miR-34a (ID000426)\ or *RNU6B* (endogenous control as recommended by Applied Biosystems; ID001093) contained in the TaqMan MicroRNA Reverse Transcription kit (Applied Biosystems). The reverse transcriptase reaction was performed by incubating the samples at 16°C for 30 min, 42°C for 30 min, and 85°C for 5 min. The PCR reaction (20 µl) contained 1.3 µl of reverse transcriptase product, 10 µl of Taq-Man 2× Universal PCR Master Mix, and 1 µl of the appropriate TaqMan MicroRNA Assay (20×) containing primers and probes for the miR of interest. The PCR mixtures were incubated at 95°C for 10 min, and this was followed by 40 cycles of 95°C for 15 s and 60°C for 60 s. The expression of miRs was based on the DCT method, using RNU6B as an endogenous control.

For the measurement of the *CDK5R1* transcript from total RNA or from polysomal/subpolysomal fractions, total cDNA was synthesized using the High Capacity cDNA Reverse Transcription Kit (Applied Biosystems). Real-Time PCR was performed using Kapa SYBR fast qPCR master mix (Kapa Biosystems, Woburn, MA, USA) and primers *CDK5R1* fw: TGAGCGGGTCTAGTGGAAAG, CDK5R1 *rev: AGCAGCAGACAAGGGGGTAG, GAPDH fw: TGCACCACCAACTGCTTAGC, GAPDH rev: GGCATGGACTGTGGTCATGAG*, *Alu* fw: GAGGCTGAGGCAGGAGAATCG and *Alu* rev: GTCGCCCAGGCTGGAGTG. The PCR reaction (10 µl) contained 1 µl of reverse transcriptase product, 5 µl of TaqMan 2× Universal PCR Master Mix, and 0.2 µl of each primer. The PCR mixtures were incubated at 95°C for 3 min, and this was followed by 40 cycles of 95°C for 10 s, 60°C for 20 s and 72°C for 10 s. The expression of *CDK5R1* was based on the DDCT method, using *GAPDH* or *Alu* as internal controls. All PCRs were performed in triplicate using an iQ5 Real Time PCR detection system (Bio-Rad).

### In vitro scratch assay

320,000 SK-N-BE cells were seeded in 30-mm diameter Petri-dishes in complete medium, they were grown to 80% confluency and then transfected with 50 nM and 100 nM pre-miR-107, pre-miR-103, *CDK5R1* siRNA and with 50/100 nM negative control siRNA and miR as previously described. After 24 h the transfection medium was replaced with fresh medium. When the cells reached the 100% confluence a scratch was made through the cell layer using a sterile micropipette tip. After washes with PBS, serum-free medium was added. The images of the wounded area were captured immediately after the scratch (T_0_) and 8 and 24 h later (T_8_ and T_24_) to monitor the cell migration into the wounded area. The migration abilities were quantified by measuring the area of the scratched regions using the ImagePro Plus 4.5 software. The experiment was performed twice.

### 3′-UTR constructs

The entire human *CDK5R1* 3′-UTR, or segments of it, were PCR-amplified from PAC144O22 using PFU Taq polymerase (Promega, San Diego, CA, USA) with proofreading activity and using primers containing flanking XbaI recognition sequence (Table 2).

**Table 2:** Oligonucleotides used to generate the 3′-UTR luciferase constructs of the human *CDK5R1* gene.

Fragment	Size (bp)	Sequence	PCR annealing temperature (°C)
UTR	2688	Fw: 5′ GC**TCTAGA**ATCGGTGAGCACTGTGCCTG 3′	62
		Rev: 5′ GC**TCTAGA**GAATCAGCACAGTACAAAAATAAAT 3′	
Fr1	662	Fw: 5′ GC**TCTAGA**ATCGGTGAGCACTGTGCCTG 3′	62
		Rev: 5′ GC**TCTAGA**AGCAGCAGACAAGGGGGTAG 3′	
Fr2	357	Fw: 5′ GC**TCTAGA**TGAGCGGGTCTAGTGGAAAG 3′	58
		Rev: 5′ GC**TCTAGA**GTATGGCATCCCTCACCTTG 3′	
Fr3	365	Fw: 5′ GC**TCTAGA**CAAGGTGAGGGATGCCATAC 3′	56
		Rev: 5′ GC**TCTAGA**GAAAGAAAATCAATAAAGTACAC 3′	
Fr4	320	Fw: 5′ GC**TCTAGA**TGCTGGAATAGGGACCTGG 3′	56
		Rev: 5′ GC**TCTAGA**GTGCTGTGTGAAGTCTGTG 3′	
Fr5	346	Fw: 5′ GC**TCTAGA**GGGACTGTCAGATAATCGGTG 3′	60
		Rev: 5′ GC**TCTAGA**TCCAGGTTTACAAGAAAAAGAGAA 3′	
Fr6	159	Fw: 5′ GC**TCTAGA**GGCTTCACACTGAGGGAACTA 3′	58
		Rev: 5′ GC**TCTAGA**GTAGGTTTTTTTTTATTGTTGATC 3′	
Fr7	213	Fw: 5′ GC**TCTAGA**TTTAGATCAACAATAAAAAAAAAC 3′	54
		Rev: 5′ GC**TCTAGA**TCCAGGTTTACAAGAAAAAGAGAA 3′	

XbaI recognition site sequence is bolded.

The PCR products were ligated in the XbaI restriction site downstream of the Renilla luciferase coding region of the pGL4.71 vector (Promega), in which the SV40 promoter region from the pGL3-Promoter vector (Promega) was previously cloned to obtain the pGL4.71P plasmid. The correct orientation of the insert was verified by direct sequencing using the Big DyeTM Terminator Cycle Sequence Ready Reaction Kit and a 3130XL ABI Prism Genetic Analyzer (Applied Biosystem).

The constructs deleted of the miR target sites were generated using the wt plasmids as templates and using two complementary specific 30 nt primers carrying the desired mutation (Table 3). We verified that the deletion of the miRNA sites did not create novel binding sites for the selected miRNAs and that no known regulatory elements were created using UTRscan at http://itbtools.ba.itb.cnr.it/utrscan and RegRNA at http://regrna.mbc.nctu.edu.tw/. A mixture with 10× buffer and 1.25 U of Pfu Turbo DNA Polymerase (Stratagene, CA, USA), 62.5 ng of each primer, 0.4 mM dNTPs and 5–50 ng template plasmid was incubated at 95°C for 30 s followed by 18 cycles at 95°C for 30 s, 55°C for 1 min and 68°C for 2 min per Kb plasmid length. Then the 5 U DpnI enzyme (Fermentas, St. Leon-Rot, Germany) for 5 h at 37°C were used to degrade the template plasmid, which is methylated because of bacterial origin.

**Table 3:** Oligonucleotides used to delete miR-103/107 target sites.

Name	Sequence
delSite1	Fw: 5′ GGTCAGGGTAGGCAACCCTGTATGGAGCT 3′
	Rev: 5′ AGCTCCATACAGGGTTGCCTACCCTGGACC 3′
delSite2/3	Fw: 5′ GCCCCCCTACCCCCTCCCAGCCACGTTGT 3′
	Rev: 5′ ACCAACGTGGCTGGGAGGGGGTAGGGGGGC 3′
delSite4	Fw: 5′ AGGGCTAAGGCCTCTGCACATG 3′
	Rev: 5′ ACAAATGTCATGTGCAGAGGCCTTAGCCCT 3′
delSite5/6	Fw: 5′ CTACAACTGATCATTTTTGGTTTCCACCTT 3′
	Rev: 5′ AAGGTGGAAACCAAAAATGATCAGTTGTAG 3′
delSite7	Fw: 5′ GGAGGACATCTGCCTCCTGAGGCCCAGCAG 3′
	Rev: 5′ CTGCTGGGCCTCAGGAGGCAGATGTTCTCC 3′
delSite8	Fw: 5′ GCGGTGGAAGGAGCCGGCACAGACTTCACA 3′
	Rev: 5′ TGTGAAGTCTGTGCCGGCTCCTTCCACCGC 3′
delSite9	Fw: 5′ CAGCCTGTCTTCAGAGCGTGCGACCAGAGG 3′
	Rev: 5′ CCTCTGGTCGCACGCTCTGAAGACAGGCTG 3′
delSite10	Fw: 5′ AGAACCCATTTGCCAATGAACACTACTTTT 3′
	Rev: 5′ AAAAGTAGTCTTCATTGGCAAATGGTGTTCT 3′
delSite11	Fw: 5′ CCTTATTCTTTAATTTTACGGTGATATTG 3′
	Rev: 5′ CCAATATCACCGTAAAATTAAAGAATAAAGG 3′

### Luciferase assay

For luciferase activity assays, 1.3×10^5^ SK-N-BE cells per well were seeded in 12-well dishes in 1 ml of medium 24 h before transfection. Cells were transfected at 60–70% confluence using 2 µl of Lipofectamine 2000 (Invitrogen) with 150 ng of the pGL4.71P constructs containing different CDK5R1 3′-UTR fragments. Transfection efficiency was >90% as measured by cell counting after transfection of a GFP expressing plasmid. To normalize the value of Renilla luciferase activity for transfection efficiency and cell viability after transfection, the pGL3-Promoter Firefly luciferase reporter gene was cotransfected (150 ng). 24 h after transfection cells were washed with PBS and lysed with passive lysis buffer (Promega), the Renilla and Firefly luciferase activity were measured using the Dual-Glo Luciferase Assay System (Promega) in a single channel luminometer.

For transient transfection of SK-N-BE cells with pre-miRs and luciferase constructs, 3.5×10^5^ cells were seeded in 6-well plates and then transfected with 100 nm double-stranded hsa-miRs, 24 h after seeding, and with the luciferase reporter constructs (300 ng), 48 h after seeding, using Lipofectamine 2000 transfection reagent according to the manufacturer's instruction. 72 h after seeding, the luciferase activity was determined as described above. Each experiment was performed at least three times.

### Statistical analysis

Histograms represent the mean values, and bars indicate standard deviation of the mean. The statistical significance of the results was determined using Student's t-test, with data considered significant when *p*<0.05. The degree of linear relationship between miRNAs and p35 expression was calculated using Pearson's correlation coefficient using the mean values of three experiments.

## Supporting Information

Figure S1 **Conservation of miR-103 target sites and hybridization analysis with** ***CDK5R1*** **3′-UTR.** Conservation of the predicted miR-103 target sites in the 3′-UTR of human *CDK5R1* (RefSeq Accession Number NM_003885) comparing five different species (hs, *Homo sapiens*; pt, *Pan troglodytes*; mm, *Mus musculus*; rt; *Rattus norvegicus*; cf, *Canis familiaris*) and prediction of the mininum free energy hybridization (mfe) of microRNA/target duplexes assessed by the RNAhybrid program.
(DOC)

Figure S2 **Conservation of miR-107 target sites and hybridization analysis with** ***CDK5R1*** **3′-UTR.** Conservation of the predicted miR-107 target sites in the 3′-UTR of human *CDK5R1* (RefSeq Accession Number NM_003885) comparing five different species (hs, *Homo sapiens*; pt, *Pan troglodytes*; mm, *Mus musculus*; rt; *Rattus norvegicus*; cf, *Canis familiaris*) and prediction of the mininum free energy hybridization (mfe) of microRNA/target duplexes assessed by the RNAhybrid program.
(DOC)

Figure S3 **Silencing of p35 by** ***CDK5R1*** **siRNA.** p35 amounts detected by western blot in *CDK5R1* siRNA, negative control (C^−^ siRNA) and untransfected SK-N-BE cells. **A**) The p35 protein levels are decreased by 41% (**p<0.01) and 38% (*p<0.05) in SK-N-BE transfected with 50 nM and 100 nM siRNA p35, respectively, 48 h after transfection, compared to untransfected cells. **B**) The p35 protein levels are decreased by 42% and 35% (**p<0.01) in SK-N-BE transfected with 50 nM and 100 nM *CDK5R1* siRNA, respectively, 72 h after transfection, compared to untransfected cells.
(TIF)
