# Supplementary material for: The Role of miR-103 and miR-107 in Regulation of CDK5R1 Expression and in Cellular Migration
Source: PLoS One. 2011 May 23;6(5):e20038. doi: 10.1371/journal.pone.0020038 (PMC3100319; doi:10.1371/journal.pone.0020038)

>hsa-miR-103

AGCAGCAUUGUACAGGGCUAUGA

Site 1

hs_NM_003885.0 UGCUGGGUCCAGGGUAGGCAAGGCUGCCGGCUGCACC

pt_NM_003885.0 UGCUGGGUCCAGGGUAGGCAAGGCUGCCGGCUGCACC

mm_NM_003885.0 CGAUGGAUCCAGGGUAGAUGAGGCUUCCCACUGUCCC

rn_NM_003885.0 CGAUGGAUCCAGGGUAGAUGAGGCUUCCCGCUGUCCC

cf_NM_003885.0 UGCUGGA-CCAGGGCAGGUGAGGCUGCCCGCCGCUCC

* *** ****** ** ***** ** * * **

**TARGET : hs_NM_003885**

**MIRNA : hsa-miR-103**

**mfe**: -25.3 kcal/mol

**position 2**

target 5' G AGG AG AG C 3'

GGUCC GU GCA GCUGC

UCGGG CA UGU CGACG

miRNA 3' AGUA A UA A 5'


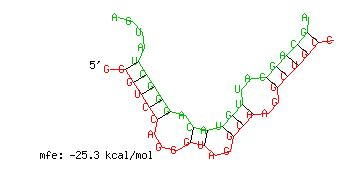


Site 2/3

hs_NM_003885.0 AACUCGCCCCCCUACCCCCUUGUCUGCUGCUCCCAGCCACG

pt_NM_003885.0 AACUCGCCCCCCUACCCCCUUGUCUGCUGCUCCCAGCCACG

mm_NM_003885.0 AGCUC-CCCUCCCAUCCUUUUGUCU-CUGCUC----CCACG

rn_NM_003885.0 AGCUC-CCUCCCCGUCCUUUUGUCU-GUGCU-----CCACC

cf_NM_003885.0 AACUUGCUCCCUGGCCUCCUUACCCCCUGCUCCCAGCCGUG

* ** * * * ** * **** **

**TARGET : hs_NM_003885**

**MIRNA : hsa-miR-103**

**mfe**: -20.5 kcal/mol

**position 1**

target 5' C CCC CCCCU U 3'

GCCC UAC UG CUGCU

CGGG AUG AC GACGA

miRNA 3' AGUAU AC UU 5'


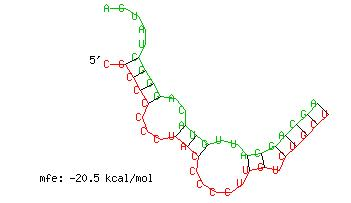


**TARGET : hs_NM_003885**

**MIRNA : hsa-miR-103**

**mfe**: -22.1 kcal/mol

**position 10**

target 5' C C 3'

CCUUGU UGCUGCU

GGGACA ACGACGA

miRNA 3' AGUAUC UGUU 5'


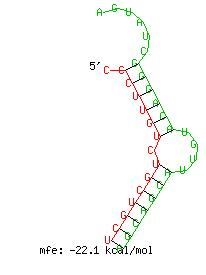


Site 4

hs_NM_003885.0 ACCUAAGGGCUAAGGCCUCUUGCUGGUGCACAUGACAU

pt_NM_003885.0 ACCUAAGAGCUAAGGCCUCUUGCUGGUGCACAUGACAU

mm_NM_003885.0 ACUUGCAUGCUAAGGCCUCUCGCUGGUGCCCGUGAUCU

rn_NM_003885.0 ACUUACAUGCUAAGGCCUCUUGCUGGUGCACAUGAUAC

cf_NM_003885.0 ACCUGAGGGCUAAGGCCUCUUGCUGGUG--CAUGACAU

** * ************ ******* * ***

**TARGET : hs_NM_003885**

**MIRNA : hsa-miR-103**

**mfe**: -19.2 kcal/mol

**position 3**

target 5' G AA GC UCU G G 3'

GGCU G C UGCUG U

UCGG C G ACGAC A

miRNA 3' AGUA GA AU UU G 5'


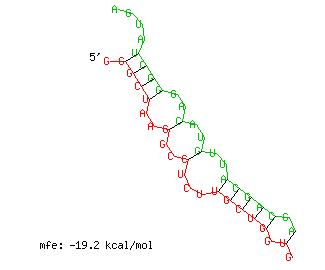


Site 6

hs_NM_003885.0 UUCUACAACUGAUCAUUUGCAGCUGCUGGUUUUGGUU-UC

pt_NM_003885.0 UUCUACAACUGAUCAUUUGCAGCUGCUGGUUUUGGUU-UC

mm_NM_003885.0 UG-UGCAGCUGAGCACUUCCAGCUGCAGGGUGUGGCCGCC

rn_NM_003885.0 UG-UCCAGCUGAUCACUUCCAGCUGCUGGGUGUGGUG-CC

cf_NM_003885.0 UC-UGCACCUGAUCAUUUGCAGCUGCCGAUUUGGGUU-UC

* * ** **** ** ** ******* * * ** *

**TARGET : hs_NM_003885**

**MIRNA : hsa-miR-103**

**mfe**: -18.2 kcal/mol

**position 1**

target 5' A AUCAUU G 3'

CUG UGCA GCUGCU

GAC AUGU CGACGA

miRNA 3' AGUAUCGG UA 5'


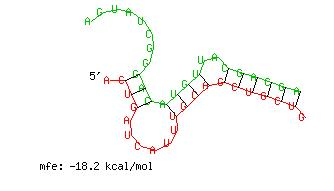


Site 7

hs_NM_003885.0 G--CCGGAGAACAUCUGCCUUG--CUGCACCUGAGGCCC

pt_NM_003885.0 G--CGGGAGAGCAUCUGCCUUG--CUGCACCUGAGGCCC

mm_NM_003885.0 A--CUAGAGAGCCUUUCCCCGGCCCCUCAUCCGGGUUCC

rn_NM_003885.0 G-ACUAGAGGGCCUUUCCCCAGUCCCUCAUCCGGGUUCC

cf_NM_003885.0 GGGCUGGGAUGGCGAUCCCUCG--CCCCAGCUGCGCU--

* * * ** * * ** * * *

**TARGET : hs_NM_003885**

**MIRNA : hsa-miR-103**

**mfe**: -21.5 kcal/mol

**position 7**

target 5' A CU A 3'

CAU GCCU UGCUGC

GUA CGGG ACGACG

miRNA 3' A U ACAUGUU A 5'


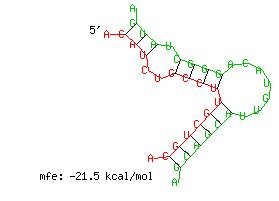


Site 8

hs_NM_003885.0 CAGCGGUGGAAGGAGCCUGC---GGCUGCUG-GCACAGACU

pt_NM_003885.0 CAGCGGUGGAAGGAGCCUGC---GGCUGCUG-GCACAGACU

mm_NM_003885.0 CGGAGCUGGAAAGC-CCUGCAAGGCUUGCUGAGUGCAGGCC

rn_NM_003885.0 UGGAGCUGGAAAGC-CCUGCAAGGCCUGCUGACUACAGGCC

cf_NM_003885.0 CGGAGCUGGAAGGAACCU-C---GGCUGCUG-CCACAGACU

* * ***** * *** * * ***** *** *

**TARGET : hs_NM_003885**

**MIRNA : hsa-miR-103**

mfe: -23.9 kcal/mol

**position 9**

target 5' G G G 3'

AGCCU GC GCUGCU

UCGGG UG CGACGA

miRNA 3' AGUA ACA UUA 5'


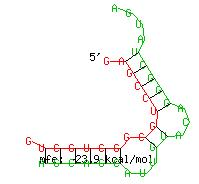


Site 9

hs_NM_003885.0 CACAGCCUGUC--UUCAGAUCCUGCUGCCGCGUGCGACC

pt_NM_003885.0 CACAGCCUGUC--UUCAGAUCCUGCUGCCGCGUGCGACC

mm_NM_003885.0 UGCAGCCCAUC--CUCACAUCCUGCUGCCACG-GUGACC

rn_NM_003885.0 UGCAGCCCAUC--CUCAGAUCCUGGAGCCCCC----CCC

cf_NM_003885.0 CCCAGCCCGUCUGUUCAGAUCCCGCUGCCACGUGUGACC

***** ** *** **** * *** * **

**TARGET : hs_NM_003885**

**MIRNA : hsa-miR-103**

**mfe**: -22.1 kcal/mol

**position 1**

target 5' G CUU AUCC C 3'

CCUGU CAG UGCUGC

GGACA GUU ACGACG

miRNA 3' AGUAUCG U A 5'


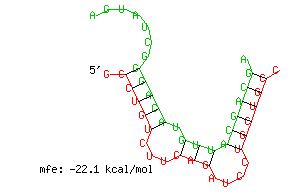


Site 10

hs_NM_003885.0 AGCAAAGAACCCAUUUGCCAUGCUGCUAUGAAGACUA

pt_NM_003885.0 AGCAAAGAACCCAUUUGCCAUGCUGCUAUGAAGACUA

mm_NM_003885.0 AGCAAAGAACCUAUCUGACAUGCUGCUAUGAAGAC-A

rn_NM_003885.0 AGCAAAGAACCCGUCUGACAUGCUGCUAUGAAGAC-A

cf_NM_003885.0 GGCCCUGAGCCCAUCUGACAUGCUGCUAUGAAGACUA

** ** ** * ** ***************** *

**TARGET : hs_NM_003885**

**MIRNA : hsa-miR-103**

**mfe**: -24.1 kcal/mol

**position 5**

target 5' A AUU C A 3'

CCC UGC AUGCUGCU

GGG AUG UACGACGA

miRNA 3' AGUAUC AC U 5'


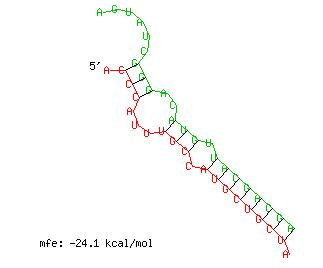


Site 11

hs_NM_003885.0 AAAACC--UUUAUUCUUUAAUUGUUGCU

pt_NM_003885.0 AAAACC--UUUAUUCUUUAAUUCUUGCU

mm_NM_003885.0 AACCCC--UUUAUUC-----UUGUUGCU

rn_NM_003885.0 AACCCCCUUUUAUUC-----UUGUUGCU

cf_NM_003885.0 AACCCC--UUUAUUCUUUAAUUGUUGCG

gg_NM_003885.0 CAUUCC--UCCCUCCUCUCAUCGUUGCU

* ** * * * * ****

**TARGET : hs_NM_003885**

**MIRNA : hsa-miR-103**

**mfe**: -16.7 kcal/mol

**position 2**

target 5' A U UCUUUAAU 3'

CCU UAU UGUUGCU

GGA AUG ACGACGA

miRNA 3' AGUAUCG C UU 5'


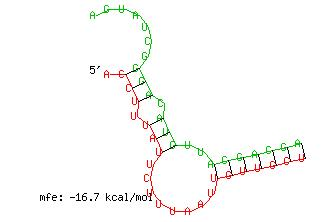

Supplement: Figure S1 — Conservation of miR-103 target sites and hybridization analysis with CDK5R1 3′-UTR. Conservation of the predicted miR-103 target sites in the 3′-UTR of human CDK5R1 (RefSeq Accession Number NM_003885) comparing five different species (hs, Homo sapiens; pt, Pan troglodytes; mm, Mus musculus; rt; Rattus norvegicus; cf, Canis familiaris) and prediction of the mininum free energy hybridization (mfe) of microRNA/target duplexes assessed by the RNAhybrid program. (DOC) [file pone.0020038.s001.doc]
