# Supplementary figures and images for: The Role of miR-103 and miR-107 in Regulation of CDK5R1 Expression and in Cellular Migration
Source: PLoS One. 2011 May 23;6(5):e20038. doi: 10.1371/journal.pone.0020038 (PMC3100319; doi:10.1371/journal.pone.0020038)

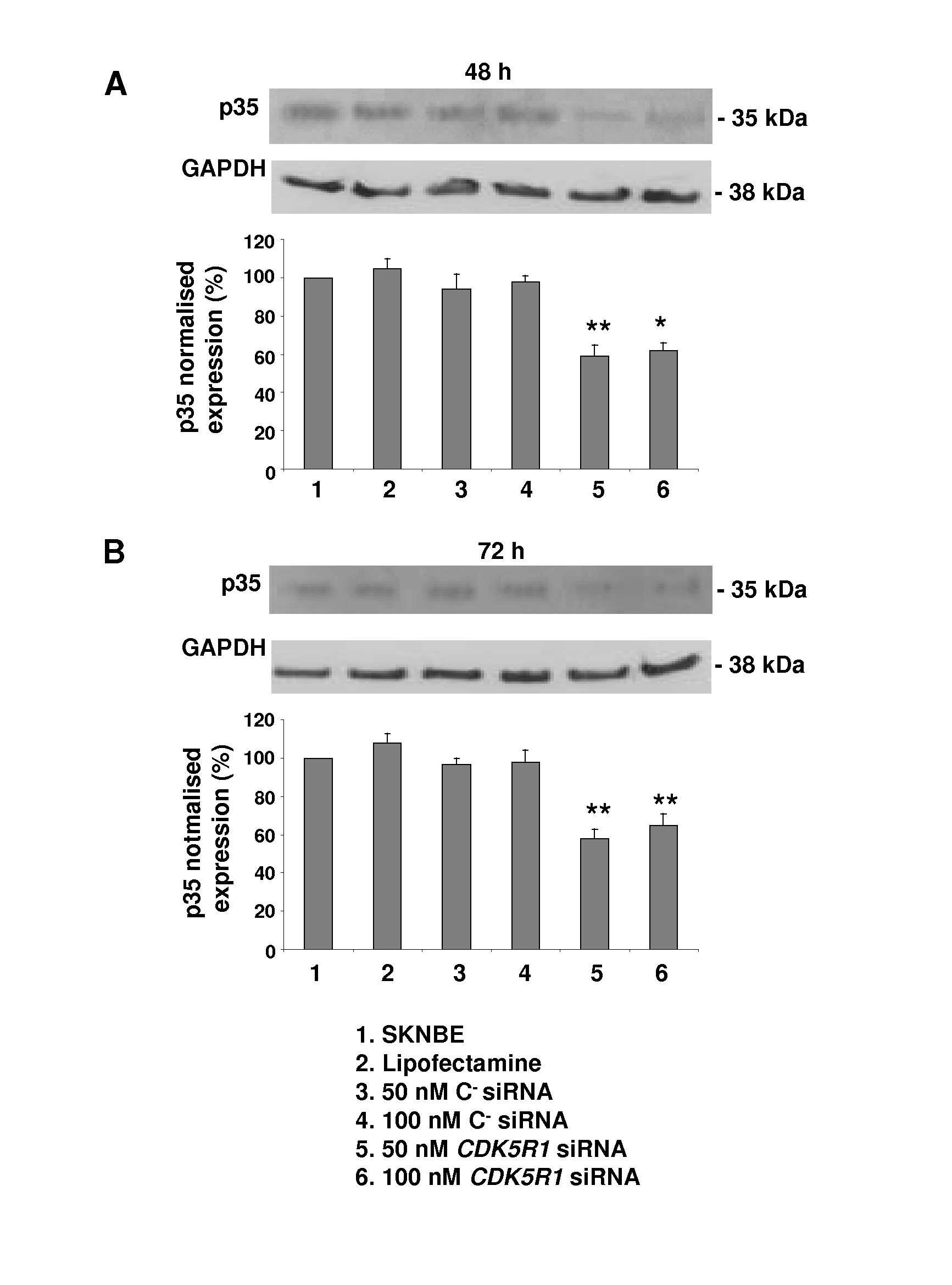

Supplement: Figure S3 — Silencing of p35 by CDK5R1 siRNA. p35 amounts detected by western blot in CDK5R1 siRNA, negative control (C− siRNA) and untransfected SK-N-BE cells. A) The p35 protein levels are decreased by 41% (**p<0.01) and 38% (*p<0.05) in SK-N-BE transfected with 50 nM and 100 nM siRNA p35, respectively, 48 h after transfection, compared to untransfected cells. B) The p35 protein levels are decreased by 42% and 35% (**p<0.01) in SK-N-BE transfected with 50 nM and 100 nM CDK5R1 siRNA, respectively, 72 h after transfection, compared to untransfected cells. (TIF) [file pone.0020038.s003.tif]
